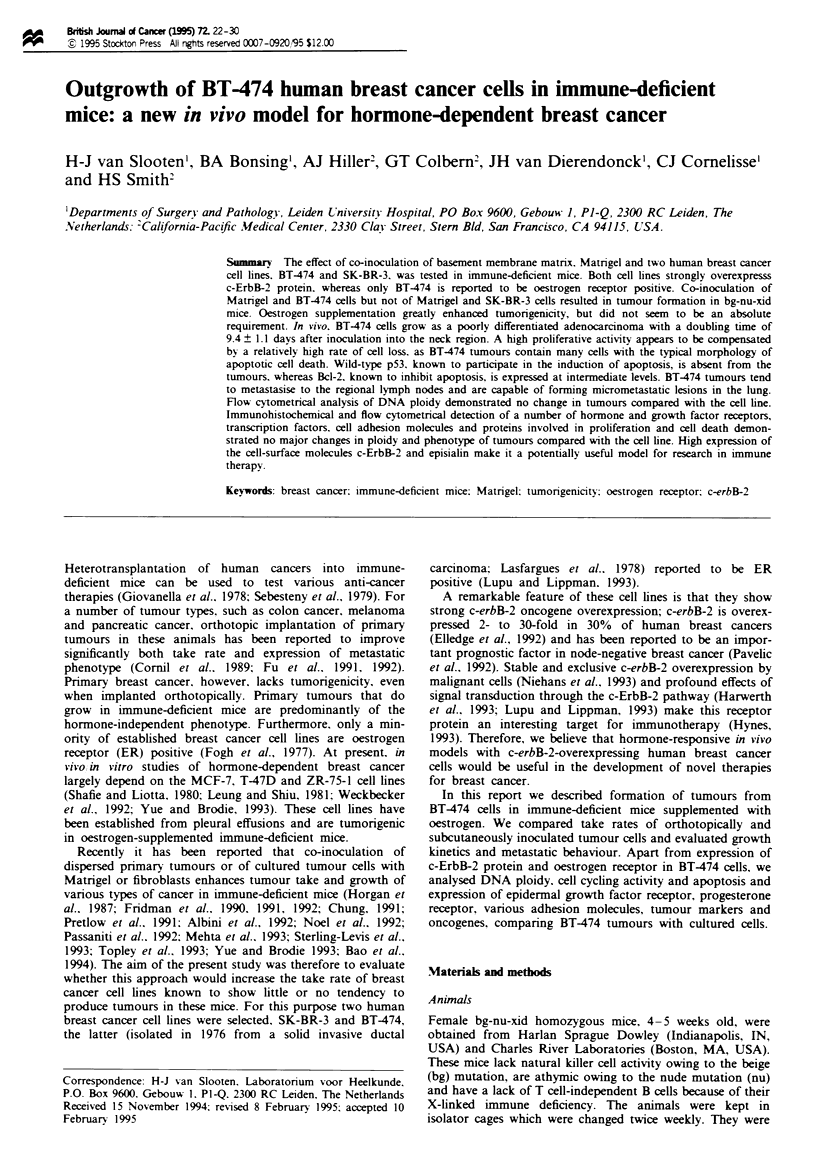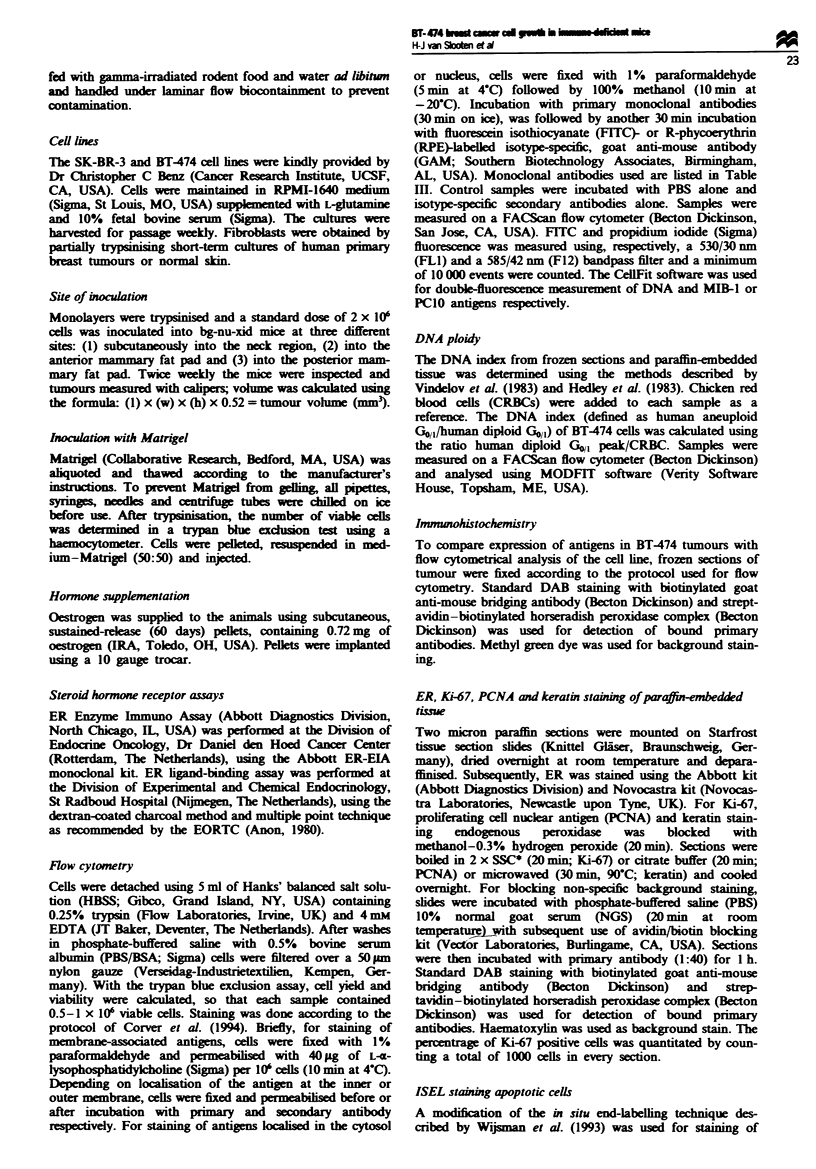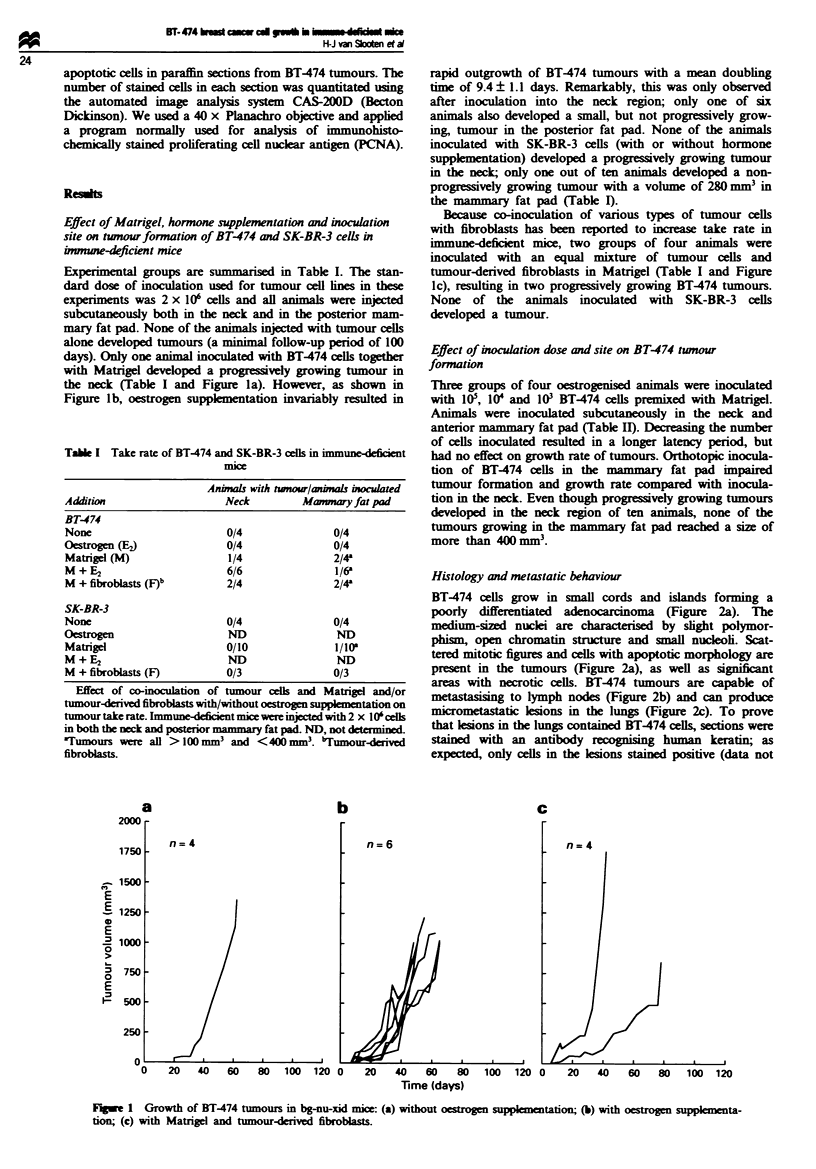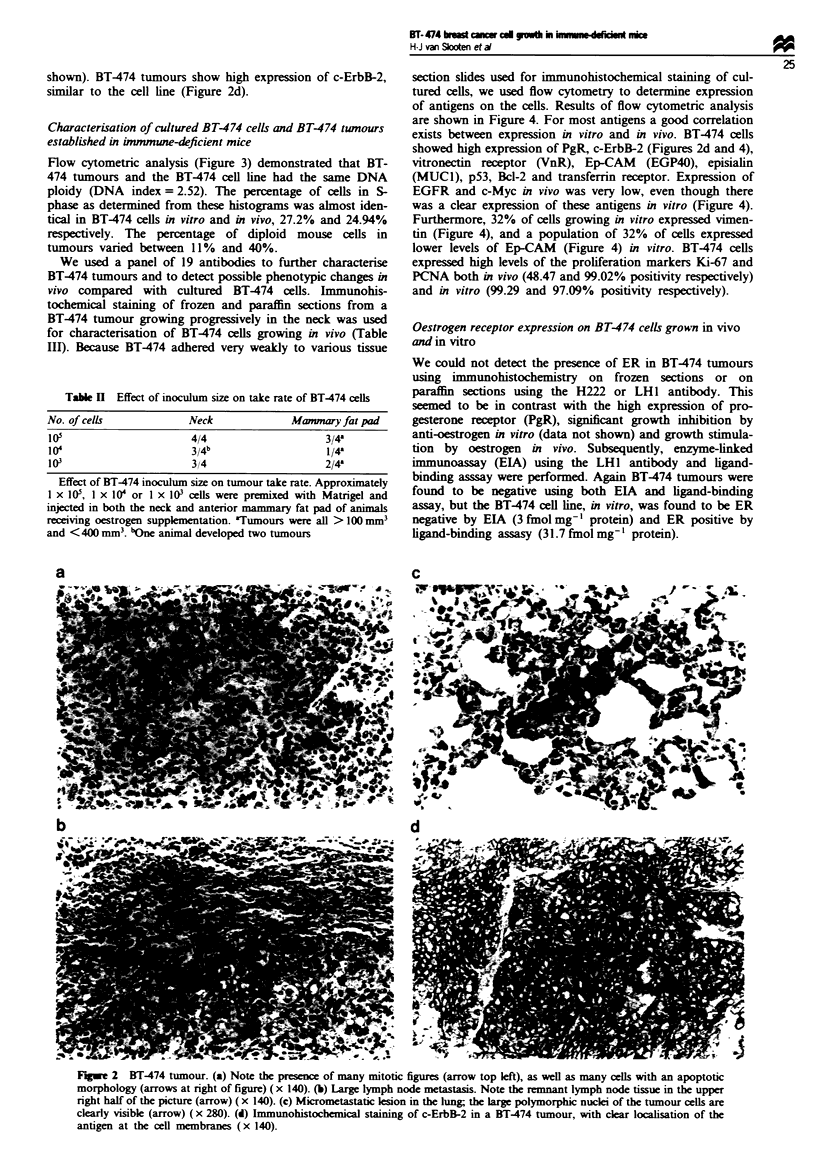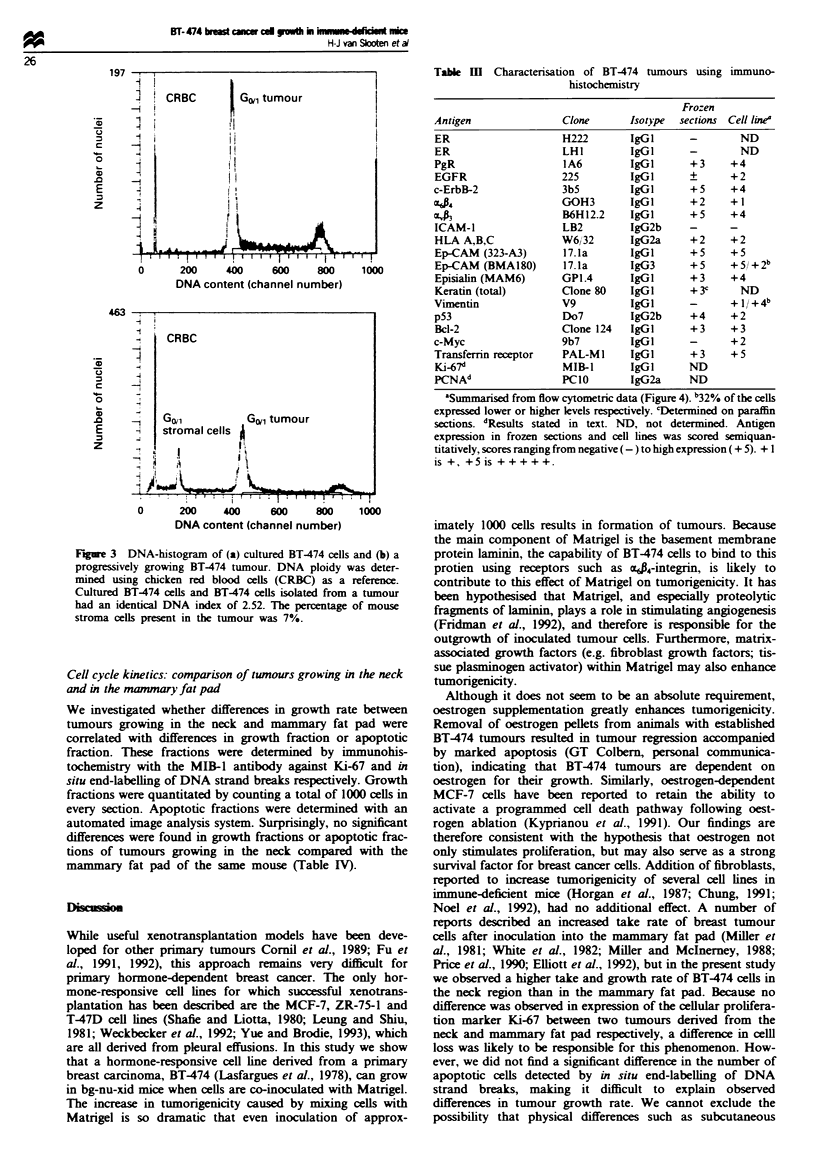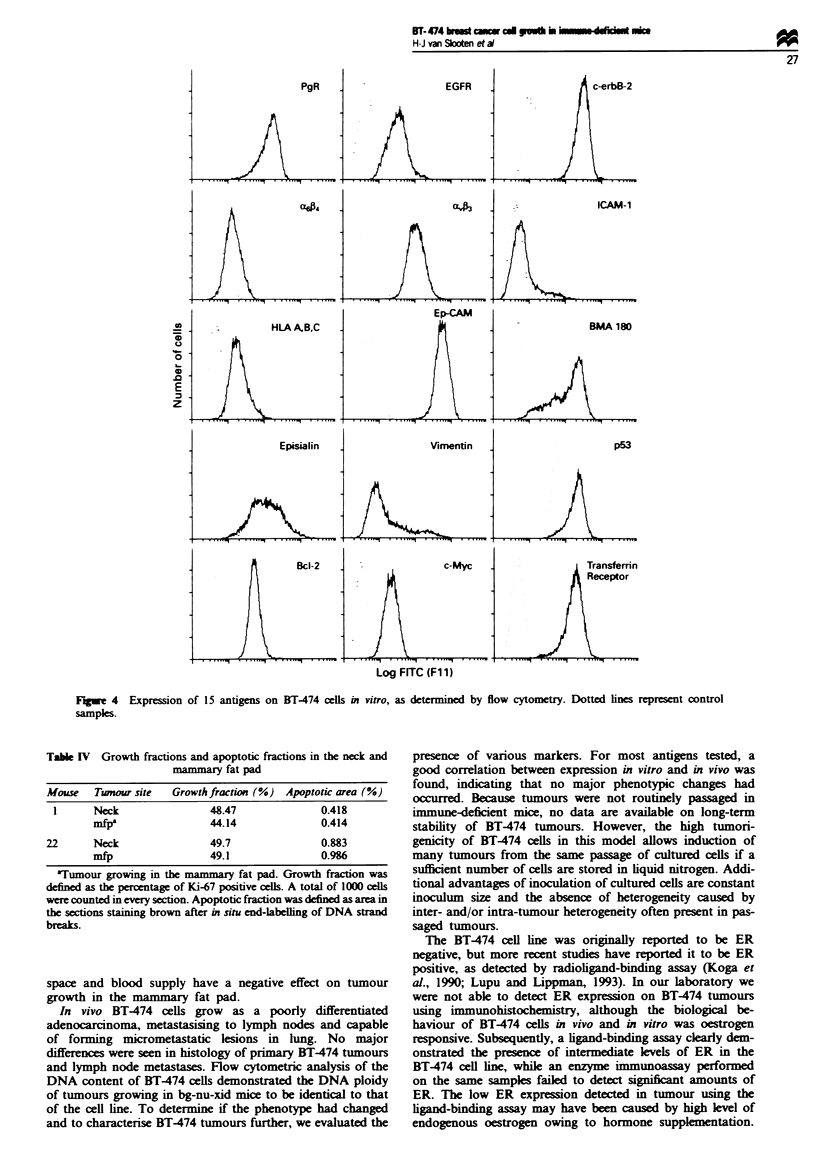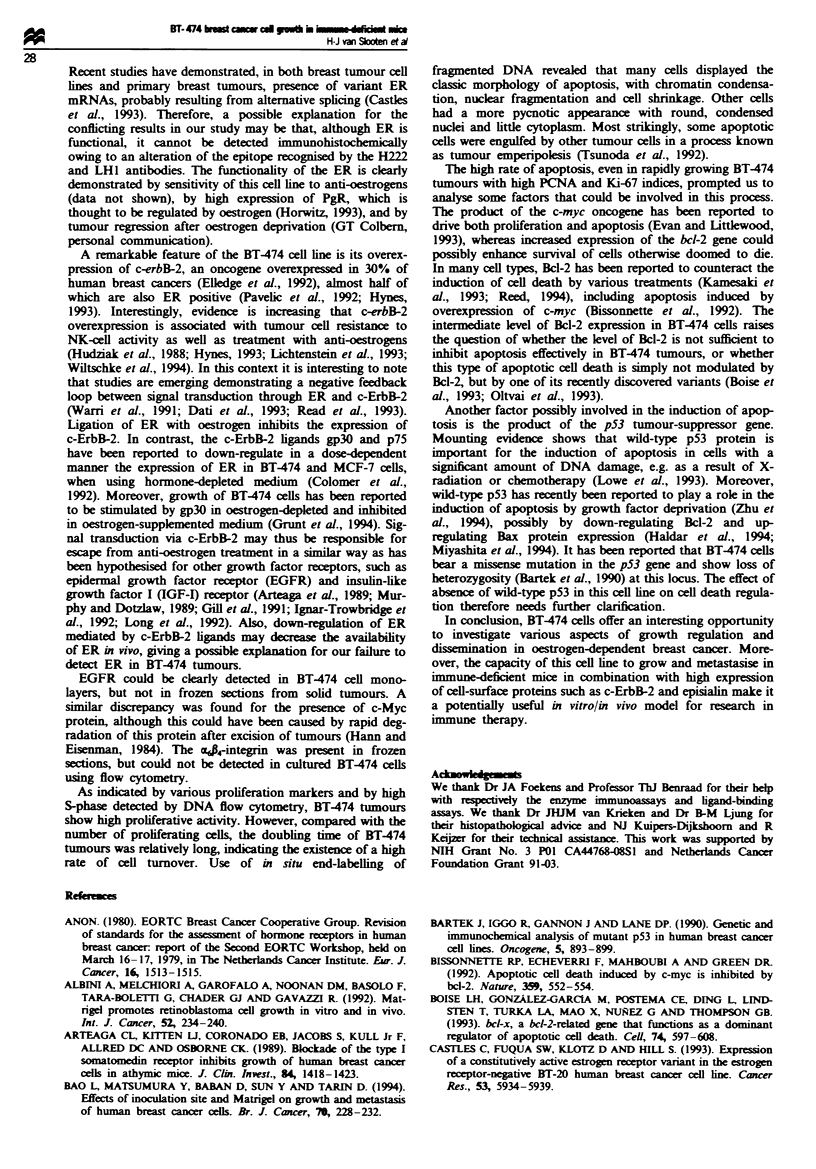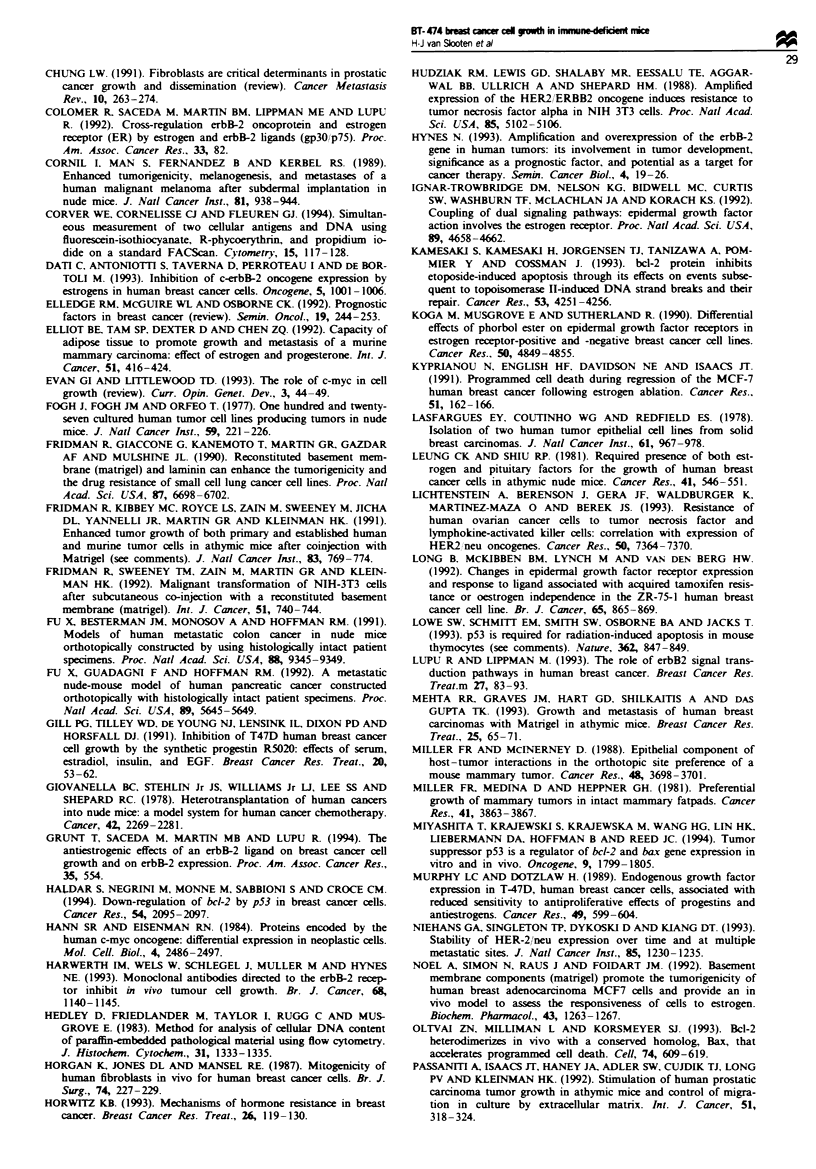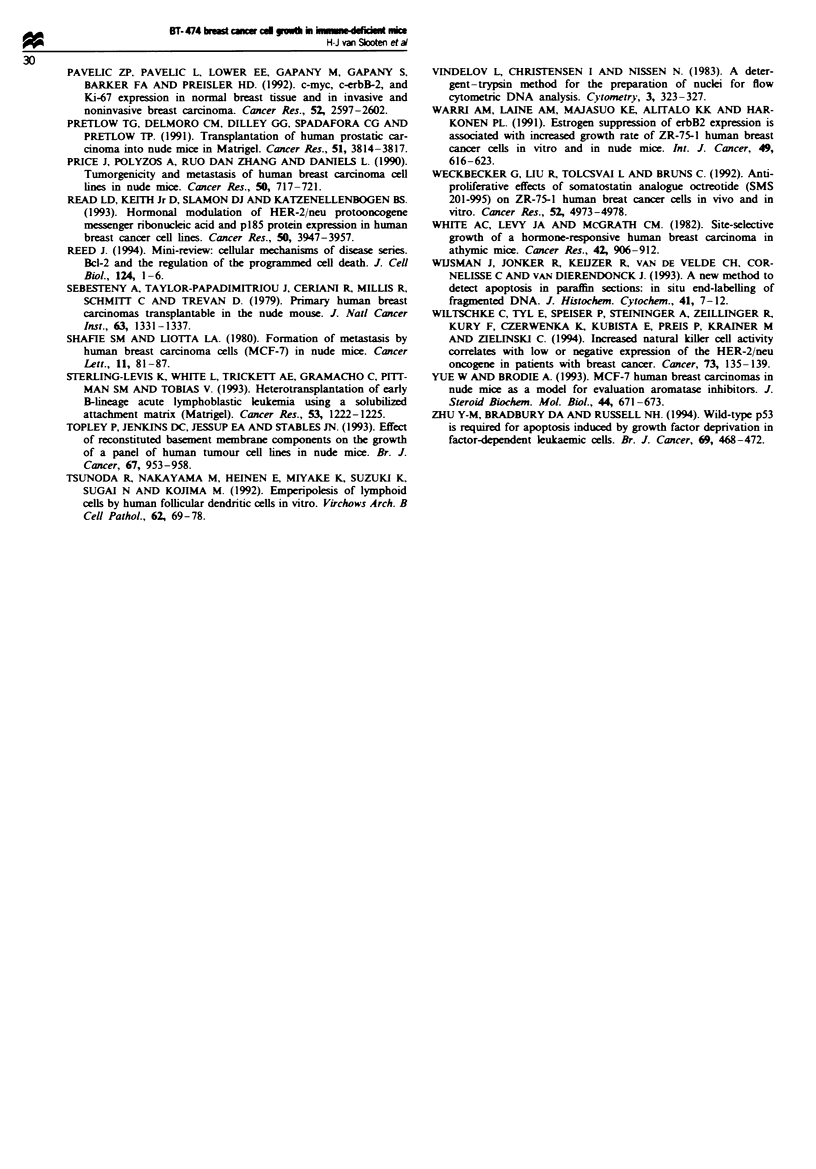# Outgrowth of BT-474 human breast cancer cells in immune-deficient mice: a new in vivo model for hormone-dependent breast cancer.

**DOI:** 10.1038/bjc.1995.271

**Published:** 1995-07

**Authors:** H. J. van Slooten, B. A. Bonsing, A. J. Hiller, G. T. Colbern, J. H. van Dierendonck, C. J. Cornelisse, H. S. Smith

**Affiliations:** Department of Surgery, Leiden University Hospital, The Netherlands.

## Abstract

**Images:**


© 1995 Stockton Press All rights reserved 0007-0920/95 $12.00

### Outgrowth of BT-474 human breast cancer cells in immune-deficient mice: a new *in vivo* model for hormone-dependent breast cancer

H-J van Slooten[1], BA Bonsing[1], AJ Hiller[2], GT Colbern[2], JH van Dierendonck[1], CJ Cornelisse[1] and HS Smith[2]

[1]*Departments of Surgery and Pathology, Leiden University Hospital, PO Box 9600, Gebouw 1, P1-Q, 2300 RC Leiden, The Netherlands;* [2]*California-Pacific Medical Center, 2330 Clay Street, Stern Bld, San Francisco, CA 94115, USA.*

**Summary** The effect of co-inoculation of basement membrane matrix, Matrigel and two human breast cancer cell lines, BT-474 and SK-BR-3, was tested in immune-deficient mice. Both cell lines strongly overexpresss c-ErbB-2 protein, whereas only BT-474 is reported to be oestrogen receptor positive. Co-inoculation of Matrigel and BT-474 cells but not of Matrigel and SK-BR-3 cells resulted in tumour formation in bg-nu-xid mice. Oestrogen supplementation greatly enhanced tumorigenicity, but did not seem to be an absolute requirement. *In vivo*, BT-474 cells grow as a poorly differentiated adenocarcinoma with a doubling time of 9.4 ± 1.1 days after inoculation into the neck region. A high proliferative activity appears to be compensated by a relatively high rate of cell loss, as BT-474 tumours contain many cells with the typical morphology of apoptotic cell death. Wild-type p53, known to participate in the induction of apoptosis, is absent from the tumours, whereas Bcl-2, known to inhibit apoptosis, is expressed at intermediate levels. BT-474 tumours tend to metastasise to the regional lymph nodes and are capable of forming micrometastatic lesions in the lung. Flow cytometrical analysis of DNA ploidy demonstrated no change in tumours compared with the cell line. Immunohistochemical and flow cytometrical detection of a number of hormone and growth factor receptors, transcription factors, cell adhesion molecules and proteins involved in proliferation and cell death demonstrated no major changes in ploidy and phenotype of tumours compared with the cell line. High expression of the cell-surface molecules c-ErbB-2 and episialin make it a potentially useful model for research in immune therapy.

**Keywords**: breast cancer; immune-deficient mice; Matrigel; tumorigenicity; oestrogen receptor; c-*erb*B-2

Heterotransplantation of human cancers into immune-deficient mice can be used to test various anti-cancer therapies (Giovanella *et al.*, 1978; Sebesteny *et al.*, 1979). For a number of tumour types, such as colon cancer, melanoma and pancreatic cancer, orthotopic implantation of primary tumours in these animals has been reported to improve significantly both take rate and expression of metastatic phenotype (Cornil *et al.*, 1989; Fu *et al.*, 1991, 1992). Primary breast cancer, however, lacks tumorigenicity, even when implanted orthotopically. Primary tumours that do grow in immune-deficient mice are predominantly of the hormone-independent phenotype. Furthermore, only a minority of established breast cancer cell lines are oestrogen receptor (ER) positive (Fogh *et al.*, 1977). At present, *in vivo*/*in vitro* studies of hormone-dependent breast cancer largely depend on the MCF-7, T-47D and ZR-75-1 cell lines (Shafie and Liotta, 1980; Leung and Shiu, 1981; Weckbecker *et al.*, 1992; Yue and Brodie, 1993). These cell lines have been established from pleural effusions and are tumorigenic in oestrogen-supplemented immune-deficient mice.

Recently it has been reported that co-inoculation of dispersed primary tumours or of cultured tumour cells with Matrigel or fibroblasts enhances tumour take and growth of various types of cancer in immune-deficient mice (Horgan *et al.*, 1987; Fridman *et al.*, 1990, 1991, 1992; Chung, 1991; Pretlow *et al.*, 1991; Albini *et al.*, 1992; Noel *et al.*, 1992; Passaniti *et al.*, 1992; Mehta *et al.*, 1993; Sterling-Levis *et al.*, 1993; Topley *et al.*, 1993; Yue and Brodie 1993; Bao *et al.*, 1994). The aim of the present study was therefore to evaluate whether this approach would increase the take rate of breast cancer cell lines known to show little or no tendency to produce tumours in these mice. For this purpose two human breast cancer cell lines were selected, SK-BR-3 and BT-474, the latter (isolated in 1976 from a solid invasive ductal carcinoma; Lasfargues *et al.*, 1978) reported to be ER positive (Lupu and Lippman, 1993).

A remarkable feature of these cell lines is that they show strong c-*erb*B-2 oncogene overexpression; c-*erb*B-2 is overexpressed 2- to 30-fold in 30% of human breast cancers (Elledge *et al.*, 1992) and has been reported to be an important prognostic factor in node-negative breast cancer (Pavelic *et al.*, 1992). Stable and exclusive c-*erb*B-2 overexpression by malignant cells (Niehans *et al.*, 1993) and profound effects of signal transduction through the c-ErbB-2 pathway (Harwerth *et al.*, 1993; Lupu and Lippman, 1993) make this receptor protein an interesting target for immunotherapy (Hynes, 1993). Therefore, we believe that hormone-responsive *in vivo* models with c-*erb*B-2-overexpressing human breast cancer cells would be useful in the development of novel therapies for breast cancer.

In this report we described formation of tumours from BT-474 cells in immune-deficient mice supplemented with oestrogen. We compared take rates of orthotopically and subcutaneously inoculated tumour cells and evaluated growth kinetics and metastatic behaviour. Apart from expression of c-ErbB-2 protein and oestrogen receptor in BT-474 cells, we analysed DNA ploidy, cell cycling activity and apoptosis and expression of epidermal growth factor receptor, progesterone receptor, various adhesion molecules, tumour markers and oncogenes, comparing BT-474 tumours with cultured cells.

### Materials and methods

### *Animals*

Female bg-nu-xid homozygous mice, 4-5 weeks old, were obtained from Harlan Sprague Dowley (Indianapolis, IN, USA) and Charles River Laboratories (Boston, MA, USA). These mice lack natural killer cell activity owing to the beige (bg) mutation, are athymic owing to the nude mutation (nu) and have a lack of T cell-independent B cells because of their X-linked immune deficiency. The animals were kept in isolator cages which were changed twice weekly. They were

Correspondence: H-J van Slooten, Laboratorium voor Heelkunde, P.O. Box 9600, Gebouw 1, P1-Q, 2300 RC Leiden, The Netherlands
Received 15 November 1994; revised 8 February 1995; accepted 10 February 1995

fed with gamma-irradiated rodent food and water *ad libitum* and handled under laminar flow biocontainment to prevent contamination.

### *Cell lines*

The SK-BR-3 and BT-474 cell lines were kindly provided by Dr Christopher C Benz (Cancer Research Institute, UCSF, CA, USA). Cells were maintained in RPMI-1640 medium (Sigma, St Louis, MO, USA) supplemented with L-glutamine and 10% fetal bovine serum (Sigma). The cultures were harvested for passage weekly. Fibroblasts were obtained by partially trypsinising short-term cultures of human primary breast tumours or normal skin.

### *Site of inoculation*

Monolayers were trypsinised and a standard dose of $2 \times 10^6$ cells was inoculated into bg-nu-xid mice at three different sites: (1) subcutaneously into the neck region, (2) into the anterior mammary fat pad and (3) into the posterior mammary fat pad. Twice weekly the mice were inspected and tumours measured with calipers; volume was calculated using the formula: (l) × (w) × (h) × 0.52 = tumour volume ($mm^3$).

### *Inoculation with Matrigel*

Matrigel (Collaborative Research, Bedford, MA, USA) was aliquoted and thawed according to the manufacturer's instructions. To prevent Matrigel from gelling, all pipettes, syringes, needles and centrifuge tubes were chilled on ice before use. After trypsinisation, the number of viable cells was determined in a trypan blue exclusion test using a haemocytometer. Cells were pelleted, resuspended in medium–Matrigel (50:50) and injected.

### *Hormone supplementation*

Oestrogen was supplied to the animals using subcutaneous, sustained-release (60 days) pellets, containing 0.72 mg of oestrogen (IRA, Toledo, OH, USA). Pellets were implanted using a 10 gauge trocar.

### *Steroid hormone receptor assays*

ER Enzyme Immuno Assay (Abbott Diagnostics Division, North Chicago, IL, USA) was performed at the Division of Endocrine Oncology, Dr Daniel den Hoed Cancer Center (Rotterdam, The Netherlands), using the Abbott ER-EIA monoclonal kit. ER ligand-binding assay was performed at the Division of Experimental and Chemical Endocrinology, St Radboud Hospital (Nijmegen, The Netherlands), using the dextran-coated charcoal method and multiple point technique as recommended by the EORTC (Anon, 1980).

### *Flow cytometry*

Cells were detached using 5 ml of Hanks' balanced salt solution (HBSS; Gibco, Grand Island, NY, USA) containing 0.25% trypsin (Flow Laboratories, Irvine, UK) and 4 mM EDTA (JT Baker, Deventer, The Netherlands). After washes in phosphate-buffered saline with 0.5% bovine serum albumin (PBS/BSA; Sigma) cells were filtered over a 50 μm nylon gauze (Verseidag-Industrietextilien, Kempen, Germany). With the trypan blue exclusion assay, cell yield and viability were calculated, so that each sample contained $0.5-1 \times 10^6$ viable cells. Staining was done according to the protocol of Corver *et al.* (1994). Briefly, for staining of membrane-associated antigens, cells were fixed with 1% paraformaldehyde and permeabilised with 40 μg of L-α-lysophosphatidylcholine (Sigma) per $10^6$ cells (10 min at 4°C). Depending on localisation of the antigen at the inner or outer membrane, cells were fixed and permeabilised before or after incubation with primary and secondary antibody respectively. For staining of antigens localised in the cytosol or nucleus, cells were fixed with 1% paraformaldehyde (5 min at 4°C) followed by 100% methanol (10 min at −20°C). Incubation with primary monoclonal antibodies (30 min on ice), was followed by another 30 min incubation with fluorescein isothiocyanate (FITC)- or R-phycoerythrin (RPE)-labelled isotype-specific, goat anti-mouse antibody (GAM; Southern Biotechnology Associates, Birmingham, AL, USA). Monoclonal antibodies used are listed in Table III. Control samples were incubated with PBS alone and isotype-specific secondary antibodies alone. Samples were measured on a FACScan flow cytometer (Becton Dickinson, San Jose, CA, USA). FITC and propidium iodide (Sigma) fluorescence was measured using, respectively, a 530/30 nm (FL1) and a 585/42 nm (F12) bandpass filter and a minimum of 10 000 events were counted. The CellFit software was used for double-fluorescence measurement of DNA and MIB-1 or PC10 antigens respectively.

### *DNA ploidy*

The DNA index from frozen sections and paraffin-embedded tissue was determined using the methods described by Vindeløv *et al.* (1983) and Hedley *et al.* (1983). Chicken red blood cells (CRBCs) were added to each sample as a reference. The DNA index (defined as human aneuploid $G_{0/1}$/human diploid $G_{0/1}$) of BT-474 cells was calculated using the ratio human diploid $G_{0/1}$ peak/CRBC. Samples were measured on a FACScan flow cytometer (Becton Dickinson) and analysed using MODFIT software (Verity Software House, Topsham, ME, USA).

### *Immunohistochemistry*

To compare expression of antigens in BT-474 tumours with flow cytometrical analysis of the cell line, frozen sections of tumour were fixed according to the protocol used for flow cytometry. Standard DAB staining with biotinylated goat anti-mouse bridging antibody (Becton Dickinson) and streptavidin–biotinylated horseradish peroxidase complex (Becton Dickinson) was used for detection of bound primary antibodies. Methyl green dye was used for background staining.

### *ER, Ki-67, PCNA and keratin staining of paraffin-embedded tissue*

Two micron paraffin sections were mounted on Starfrost tissue section slides (Knittel Gläser, Braunschweig, Germany), dried overnight at room temperature and deparaffinised. Subsequently, ER was stained using the Abbott kit (Abbott Diagnostics Division) and Novocastra kit (Novocastra Laboratories, Newcastle upon Tyne, UK). For Ki-67, proliferating cell nuclear antigen (PCNA) and keratin staining endogenous peroxidase was blocked with methanol–0.3% hydrogen peroxide (20 min). Sections were boiled in 2 × SSC* (20 min; Ki-67) or citrate buffer (20 min; PCNA) or microwaved (30 min, 90°C; keratin) and cooled overnight. For blocking non-specific background staining, slides were incubated with phosphate-buffered saline (PBS) 10% normal goat serum (NGS) (20 min at room temperature) with subsequent use of avidin/biotin blocking kit (Vector Laboratories, Burlingame, CA, USA). Sections were then incubated with primary antibody (1:40) for 1 h. Standard DAB staining with biotinylated goat anti-mouse bridging antibody (Becton Dickinson) and streptavidin–biotinylated horseradish peroxidase complex (Becton Dickinson) was used for detection of bound primary antibodies. Haematoxylin was used as background stain. The percentrage of Ki-67 positive cells was quantitated by counting a total of 1000 cells in every section.

### *ISEL staining apoptotic cells*

A modification of the *in situ* end-labelling technique described by Wijsman *et al.* (1993) was used for staining of apoptotic cells in paraffin sections from BT-474 tumours. The number of stained cells in each section was quantitated using the automated image analysis system CAS-200D (Becton Dickinson). We used a 40 × Planachro objective and applied a program normally used for analysis of immunohistochemically stained proliferating cell nuclear antigen (PCNA).

### Results

### *Effect of Matrigel, hormone supplementation and inoculation site on tumour formation of BT-474 and SK-BR-3 cells in immune-deficient mice*

Experimental groups are summarised in Table I. The standard dose of inoculation used for tumour cell lines in these experiments was $2 \times 10^6$ cells and all animals were injected subcutaneously both in the neck and in the posterior mammary fat pad. None of the animals injected with tumour cells alone developed tumours (a minimal follow-up period of 100 days). Only one animal inoculated with BT-474 cells together with Matrigel developed a progressively growing tumour in the neck (Table I and Figure 1a). However, as shown in Figure 1b, oestrogen supplementation invariably resulted in rapid outgrowth of BT-474 tumours with a mean doubling time of $9.4 \pm 1.1$ days. Remarkably, this was only observed after inoculation into the neck region; only one of six animals also developed a small, but not progressively growing, tumour in the posterior fat pad. None of the animals inoculated with SK-BR-3 cells (with or without hormone supplementation) developed a progressively growing tumour in the neck; only one out of ten animals developed a non-progressively growing tumour with a volume of 280 mm³ in the mammary fat pad (Table I).

Because co-inoculation of various types of tumour cells with fibroblasts has been reported to increase take rate in immune-deficient mice, two groups of four animals were inoculated with an equal mixture of tumour cells and tumour-derived fibroblasts in Matrigel (Table I and Figure 1c), resulting in two progressively growing BT-474 tumours. None of the animals inoculated with SK-BR-3 cells developed a tumour.

**Table I** Take rate of BT-474 and SK-BR-3 cells in immune-deficient mice

| *Addition* | *Animals with tumour/animals inoculated* Neck | *Mammary fat pad* |
|---|---|---|
| *BT-474* | | |
| None | 0/4 | 0/4 |
| Oestrogen ($E_2$) | 0/4 | 0/4 |
| Matrigel (M) | 1/4 | 2/4[a] |
| M + $E_2$ | 6/6 | 1/6[a] |
| M + fibroblasts (F)[b] | 2/4 | 2/4[a] |
| *SK-BR-3* | | |
| None | 0/4 | 0/4 |
| Oestrogen | ND | ND |
| Matrigel | 0/10 | 1/10[a] |
| M + $E_2$ | ND | ND |
| M + fibroblasts (F) | 0/3 | 0/3 |

Effect of co-inoculation of tumour cells and Matrigel and/or tumour-derived fibroblasts with/without oestrogen supplementation on tumour take rate. Immune-deficient mice were injected with $2 \times 10^6$ cells in both the neck and posterior mammary fat pad. ND, not determined. [a]Tumours were all $>100$ mm³ and $<400$ mm³. [b]Tumour-derived fibroblasts.

### *Effect of inoculation dose and site on BT-474 tumour formation*

Three groups of four oestrogenised animals were inoculated with $10^5$, $10^4$ and $10^3$ BT-474 cells premixed with Matrigel. Animals were inoculated subcutaneously in the neck and anterior mammary fat pad (Table II). Decreasing the number of cells inoculated resulted in a longer latency period, but had no effect on growth rate of tumours. Orthotopic inoculation of BT-474 cells in the mammary fat pad impaired tumour formation and growth rate compared with inoculation in the neck. Even though progressively growing tumours developed in the neck region of ten animals, none of the tumours growing in the mammary fat pad reached a size of more than 400 mm³.

### *Histology and metastatic behaviour*

BT-474 cells grow in small cords and islands forming a poorly differentiated adenocarcinoma (Figure 2a). The medium-sized nuclei are characterised by slight polymorphism, open chromatin structure and small nucleoli. Scattered mitotic figures and cells with apoptotic morphology are present in the tumours (Figure 2a), as well as significant areas with necrotic cells. BT-474 tumours are capable of metastasising to lymph nodes (Figure 2b) and can produce micrometastatic lesions in the lungs (Figure 2c). To prove that lesions in the lungs contained BT-474 cells, sections were stained with an antibody recognising human keratin; as expected, only cells in the lesions stained positive (data not

**Figure 1** Growth of BT-474 tumours in bg-nu-xid mice: (a) without oestrogen supplementation; (b) with oestrogen supplementation; (c) with Matrigel and tumour-derived fibroblasts.

shown). BT-474 tumours show high expression of c-ErbB-2, similar to the cell line (Figure 2d).

*Characterisation of cultured BT-474 cells and BT-474 tumours established in immmune-deficient mice*

Flow cytometric analysis (Figure 3) demonstrated that BT-474 tumours and the BT-474 cell line had the same DNA ploidy (DNA index = 2.52). The percentage of cells in S-phase as determined from these histograms was almost identical in BT-474 cells *in vitro* and *in vivo*, 27.2% and 24.94% respectively. The percentage of diploid mouse cells in tumours varied between 11% and 40%.

We used a panel of 19 antibodies to further characterise BT-474 tumours and to detect possible phenotypic changes *in vivo* compared with cultured BT-474 cells. Immunohistochemical staining of frozen and paraffin sections from a BT-474 tumour growing progressively in the neck was used for characterisation of BT-474 cells growing *in vivo* (Table III). Because BT-474 adhered very weakly to various tissue section slides used for immunohistochemical staining of cultured cells, we used flow cytometry to determine expression of antigens on the cells. Results of flow cytometric analysis are shown in Figure 4. For most antigens a good correlation exists between expression *in vitro* and *in vivo*. BT-474 cells showed high expression of PgR, c-ErbB-2 (Figures 2d and 4), vitronectin receptor (VnR), Ep-CAM (EGP40), episialin (MUC1), p53, Bcl-2 and transferrin receptor. Expression of EGFR and c-Myc *in vivo* was very low, even though there was a clear expression of these antigens *in vitro* (Figure 4). Furthermore, 32% of cells growing *in vitro* expressed vimentin (Figure 4), and a population of 32% of cells expressed lower levels of Ep-CAM (Figure 4) *in vitro*. BT-474 cells expressed high levels of the proliferation markers Ki-67 and PCNA both *in vivo* (48.47 and 99.02% positivity respectively) and *in vitro* (99.29 and 97.09% positivity respectively).

**Table II** Effect of inoculum size on take rate of BT-474 cells

| *No. of cells* | *Neck* | *Mammary fat pad* |
|---|---|---|
| $10^5$ | 4/4 | 3/4[a] |
| $10^4$ | 3/4[b] | 1/4[a] |
| $10^3$ | 3/4 | 2/4[a] |

Effect of BT-474 inoculum size on tumour take rate. Approximately $1 \times 10^5$, $1 \times 10^4$ or $1 \times 10^3$ cells were premixed with Matrigel and injected in both the neck and anterior mammary fat pad of animals receiving oestrogen supplementation. [a]Tumours were all $> 100\ mm^3$ and $< 400\ mm^3$. [b]One animal developed two tumours

*Oestrogen receptor expression on BT-474 cells grown* in vivo *and* in vitro

We could not detect the presence of ER in BT-474 tumours using immunohistochemistry on frozen sections or on paraffin sections using the H222 or LH1 antibody. This seemed to be in contrast with the high expression of progesterone receptor (PgR), significant growth inhibition by anti-oestrogen *in vitro* (data not shown) and growth stimulation by oestrogen *in vivo*. Subsequently, enzyme-linked immunoassay (EIA) using the LH1 antibody and ligand-binding asssay were performed. Again BT-474 tumours were found to be negative using both EIA and ligand-binding assay, but the BT-474 cell line, *in vitro*, was found to be ER negative by EIA (3 fmol $mg^{-1}$ protein) and ER positive by ligand-binding assasy (31.7 fmol $mg^{-1}$ protein).

**Figure 2** BT-474 tumour. (**a**) Note the presence of many mitotic figures (arrow top left), as well as many cells with an apoptotic morphology (arrows at right of figure) ( × 140). (**b**) Large lymph node metastasis. Note the remnant lymph node tissue in the upper right half of the picture (arrow) ( × 140). (**c**) Micrometastatic lesion in the lung; the large polymorphic nuclei of the tumour cells are clearly visible (arrow) ( × 280). (**d**) Immunohistochemical staining of c-ErbB-2 in a BT-474 tumour, with clear localisation of the antigen at the cell membranes ( × 140).

**Figure 3** DNA-histogram of (**a**) cultured BT-474 cells and (**b**) a progressively growing BT-474 tumour. DNA ploidy was determined using chicken red blood cells (CRBC) as a reference. Cultured BT-474 cells and BT-474 cells isolated from a tumour had an identical DNA index of 2.52. The percentage of mouse stroma cells present in the tumour was 7%.

*Cell cycle kinetics: comparison of tumours growing in the neck and in the mammary fat pad*

We investigated whether differences in growth rate between tumours growing in the neck and mammary fat pad were correlated with differences in growth fraction or apoptotic fraction. These fractions were determined by immunohistochemistry with the MIB-1 antibody against Ki-67 and *in situ* end-labelling of DNA strand breaks respectively. Growth fractions were quantitated by counting a total of 1000 cells in every section. Apoptotic fractions were determined with an automated image analysis system. Surprisingly, no significant differences were found in growth fractions or apoptotic fractions of tumours growing in the neck compared with the mammary fat pad of the same mouse (Table IV).

### Discussion

While useful xenotransplantation models have been developed for other primary tumours Cornil *et al.*, 1989; Fu *et al.*, 1991, 1992), this approach remains very difficult for primary hormone-dependent breast cancer. The only hormone-responsive cell lines for which successful xenotransplantation has been described are the MCF-7, ZR-75-1 and T-47D cell lines (Shafie and Liotta, 1980; Leung and Shiu, 1981; Weckbecker *et al.*, 1992; Yue and Brodie, 1993), which are all derived from pleural effusions. In this study we show that a hormone-responsive cell line derived from a primary breast carcinoma, BT-474 (Lasfargues *et al.*, 1978), can grow in bg-nu-xid mice when cells are co-inoculated with Matrigel. The increase in tumorigenicity caused by mixing cells with Matrigel is so dramatic that even inoculation of approximately 1000 cells results in formation of tumours. Because the main component of Matrigel is the basement membrane protein laminin, the capability of BT-474 cells to bind to this protien using receptors such as $\alpha_6\beta_4$-integrin, is likely to contribute to this effect of Matrigel on tumorigenicity. It has been hypothesised that Matrigel, and especially proteolytic fragments of laminin, plays a role in stimulating angiogenesis (Fridman *et al.*, 1992), and therefore is responsible for the outgrowth of inoculated tumour cells. Furthermore, matrix-associated growth factors (e.g. fibroblast growth factors; tissue plasminogen activator) within Matrigel may also enhance tumorigenicity.

Although it does not seem to be an absolute requirement, oestrogen supplementation greatly enhances tumorigenicity. Removal of oestrogen pellets from animals with established BT-474 tumours resulted in tumour regression accompanied by marked apoptosis (GT Colbern, personal communication), indicating that BT-474 tumours are dependent on oestrogen for their growth. Similarly, oestrogen-dependent MCF-7 cells have been reported to retain the ability to activate a programmed cell death pathway following oestrogen ablation (Kyprianou *et al.*, 1991). Our findings are therefore consistent with the hypothesis that oestrogen not only stimulates proliferation, but may also serve as a strong survival factor for breast cancer cells. Addition of fibroblasts, reported to increase tumorigenicity of several cell lines in immune-deficient mice (Horgan *et al.*, 1987; Chung, 1991; Noel *et al.*, 1992), had no additional effect. A number of reports described an increased take rate of breast tumour cells after inoculation into the mammary fat pad (Miller *et al.*, 1981; White *et al.*, 1982; Miller and McInerney, 1988; Price *et al.*, 1990; Elliott *et al.*, 1992), but in the present study we observed a higher take and growth rate of BT-474 cells in the neck region than in the mammary fat pad. Because no difference was observed in expression of the cellular proliferation marker Ki-67 between two tumours derived from the neck and mammary fat pad respectively, a difference in celll loss was likely to be responsible for this phenomenon. However, we did not find a significant difference in the number of apoptotic cells detected by *in situ* end-labelling of DNA strand breaks, making it difficult to explain observed differences in tumour growth rate. We cannot exclude the possibility that physical differences such as subcutaneous

**Table III** Characterisation of BT-474 tumours using immunohistochemistry

| *Antigen* | *Clone* | *Isotype* | *Frozen sections* | *Cell line*[a] |
|---|---|---|---|---|
| ER | H222 | IgG1 | – | ND |
| ER | LH1 | IgG1 | – | ND |
| PgR | 1A6 | IgG1 | +3 | +4 |
| EGFR | 225 | IgG1 | ± | +2 |
| c-ErbB-2 | 3b5 | IgG1 | +5 | +4 |
| $\alpha_6\beta_4$ | GOH3 | IgG1 | +2 | +1 |
| $\alpha_v\beta_3$ | B6H12.2 | IgG1 | +5 | +4 |
| ICAM-1 | LB2 | IgG2b | – | – |
| HLA A,B,C | W6/32 | IgG2a | +2 | +2 |
| Ep-CAM (323-A3) | 17.1a | IgG1 | +5 | +5 |
| Ep-CAM (BMA180) | 17.1a | IgG3 | +5 | +5/+2[b] |
| Episialin (MAM6) | GP1.4 | IgG1 | +3 | +4 |
| Keratin (total) | Clone 80 | IgG1 | +3[c] | ND |
| Vimentin | V9 | IgG1 | – | +1/+4[b] |
| p53 | Do7 | IgG2b | +4 | +2 |
| Bcl-2 | Clone 124 | IgG1 | +3 | +3 |
| c-Myc | 9b7 | IgG1 | – | +2 |
| Transferrin receptor | PAL-M1 | IgG1 | +3 | +5 |
| Ki-67[d] | MIB-1 | IgG1 | ND | |
| PCNA[d] | PC10 | IgG2a | ND | |

[a]Summarised from flow cytometric data (Figure 4). [b]32% of the cells expressed lower or higher levels respectively. [c]Determined on paraffin sections. [d]Results stated in text. ND, not determined. Antigen expression in frozen sections and cell lines was scored semiquantitatively, scores ranging from negative (–) to high expression (+5). +1 is +, +5 is +++++.

**Figure 4** Expression of 15 antigens on BT-474 cells *in vitro*, as determined by flow cytometry. Dotted lines represent control samples.

**Table IV** Growth fractions and apoptotic fractions in the neck and mammary fat pad

| *Mouse* | *Tumour site* | *Growth fraction (%)* | *Apoptotic area (%)* |
|---|---|---|---|
| 1 | Neck | 48.47 | 0.418 |
| | mfp[a] | 44.14 | 0.414 |
| 22 | Neck | 49.7 | 0.883 |
| | mfp | 49.1 | 0.986 |

[a]Tumour growing in the mammary fat pad. Growth fraction was defined as the percentage of Ki-67 positive cells. A total of 1000 cells were counted in every section. Apoptotic fraction was defined as area in the sections staining brown after *in situ* end-labelling of DNA strand breaks.

space and blood supply have a negative effect on tumour growth in the mammary fat pad.

*In vivo* BT-474 cells grow as a poorly differentiated adenocarcinoma, metastasising to lymph nodes and capable of forming micrometastatic lesions in lung. No major differences were seen in histology of primary BT-474 tumours and lymph node metastases. Flow cytometric analysis of the DNA content of BT-474 cells demonstrated the DNA ploidy of tumours growing in bg-nu-xid mice to be identical to that of the cell line. To determine if the phenotype had changed and to characterise BT-474 tumours further, we evaluated the presence of various markers. For most antigens tested, a good correlation between expression *in vitro* and *in vivo* was found, indicating that no major phenotypic changes had occurred. Because tumours were not routinely passaged in immune-deficient mice, no data are available on long-term stability of BT-474 tumours. However, the high tumorigenicity of BT-474 cells in this model allows induction of many tumours from the same passage of cultured cells if a sufficient number of cells are stored in liquid nitrogen. Additional advantages of inoculation of cultured cells are constant inoculum size and the absence of heterogeneity caused by inter- and/or intra-tumour heterogeneity often present in passaged tumours.

The BT-474 cell line was originally reported to be ER negative, but more recent studies have reported it to be ER positive, as detected by radioligand-binding assay (Koga *et al.*, 1990; Lupu and Lippman, 1993). In our laboratory we were not able to detect ER expression on BT-474 tumours using immunohistochemistry, although the biological behaviour of BT-474 cells *in vivo* and *in vitro* was oestrogen responsive. Subsequently, a ligand-binding assay clearly demonstrated the presence of intermediate levels of ER in the BT-474 cell line, while an enzyme immunoassay performed on the same samples failed to detect significant amounts of ER. The low ER expression detected in tumour using the ligand-binding assay may have been caused by high level of endogenous oestrogen owing to hormone supplementation.

Recent studies have demonstrated, in both breast tumour cell lines and primary breast tumours, presence of variant ER mRNAs, probably resulting from alternative splicing (Castles et al., 1993). Therefore, a possible explanation for the conflicting results in our study may be that, although ER is functional, it cannot be detected immunohistochemically owing to an alteration of the epitope recognised by the H222 and LH1 antibodies. The functionality of the ER is clearly demonstrated by sensitivity of this cell line to anti-oestrogens (data not shown), by high expression of PgR, which is thought to be regulated by oestrogen (Horwitz, 1993), and by tumour regression after oestrogen deprivation (GT Colbern, personal communication).

A remarkable feature of the BT-474 cell line is its overexpression of c-*erb*B-2, an oncogene overexpressed in 30% of human breast cancers (Elledge et al., 1992), almost half of which are also ER positive (Pavelic et al., 1992; Hynes, 1993). Interestingly, evidence is increasing that c-*erb*B-2 overexpression is associated with tumour cell resistance to NK-cell activity as well as treatment with anti-oestrogens (Hudziak et al., 1988; Hynes, 1993; Lichtenstein et al., 1993; Wiltschke et al., 1994). In this context it is interesting to note that studies are emerging demonstrating a negative feedback loop between signal transduction through ER and c-ErbB-2 (Warri et al., 1991; Dati et al., 1993; Read et al., 1993). Ligation of ER with oestrogen inhibits the expression of c-ErbB-2. In contrast, the c-ErbB-2 ligands gp30 and p75 have been reported to down-regulate in a dose-dependent manner the expression of ER in BT-474 and MCF-7 cells, when using hormone-depleted medium (Colomer et al., 1992). Moreover, growth of BT-474 cells has been reported to be stimulated by gp30 in oestrogen-depleted and inhibited in oestrogen-supplemented medium (Grunt et al., 1994). Signal transduction via c-ErbB-2 may thus be responsible for escape from anti-oestrogen treatment in a similar way as has been hypothesised for other growth factor receptors, such as epidermal growth factor receptor (EGFR) and insulin-like growth factor I (IGF-I) receptor (Arteaga et al., 1989; Murphy and Dotzlaw, 1989; Gill et al., 1991; Ignar-Trowbridge et al., 1992; Long et al., 1992). Also, down-regulation of ER mediated by c-ErbB-2 ligands may decrease the availability of ER *in vivo*, giving a possible explanation for our failure to detect ER in BT-474 tumours.

EGFR could be clearly detected in BT-474 cell monolayers, but not in frozen sections from solid tumours. A similar discrepancy was found for the presence of c-Myc protein, although this could have been caused by rapid degradation of this protein after excision of tumours (Hann and Eisenman, 1984). The $\alpha_6\beta_4$-integrin was present in frozen sections, but could not be detected in cultured BT-474 cells using flow cytometry.

As indicated by various proliferation markers and by high S-phase detected by DNA flow cytometry, BT-474 tumours show high proliferative activity. However, compared with the number of proliferating cells, the doubling time of BT-474 tumours was relatively long, indicating the existence of a high rate of cell turnover. Use of *in situ* end-labelling of fragmented DNA revealed that many cells displayed the classic morphology of apoptosis, with chromatin condensation, nuclear fragmentation and cell shrinkage. Other cells had a more pycnotic appearance with round, condensed nuclei and little cytoplasm. Most strikingly, some apoptotic cells were engulfed by other tumour cells in a process known as tumour emperipolesis (Tsunoda et al., 1992).

The high rate of apoptosis, even in rapidly growing BT-474 tumours with high PCNA and Ki-67 indices, prompted us to analyse some factors that could be involved in this process. The product of the c-*myc* oncogene has been reported to drive both proliferation and apoptosis (Evan and Littlewood, 1993), whereas increased expression of the *bcl*-2 gene could possibly enhance survival of cells otherwise doomed to die. In many cell types, Bcl-2 has been reported to counteract the induction of cell death by various treatments (Kamesaki et al., 1993; Reed, 1994), including apoptosis induced by overexpression of c-*myc* (Bissonnette et al., 1992). The intermediate level of Bcl-2 expression in BT-474 cells raises the question of whether the level of Bcl-2 is not sufficient to inhibit apoptosis effectively in BT-474 tumours, or whether this type of apoptotic cell death is simply not modulated by Bcl-2, but by one of its recently discovered variants (Boise et al., 1993; Oltvai et al., 1993).

Another factor possibly involved in the induction of apoptosis is the product of the *p53* tumour-suppressor gene. Mounting evidence shows that wild-type p53 protein is important for the induction of apoptosis in cells with a significant amount of DNA damage, e.g. as a result of X-radiation or chemotherapy (Lowe et al., 1993). Moreover, wild-type p53 has recently been reported to play a role in the induction of apoptosis by growth factor deprivation (Zhu et al., 1994), possibly by down-regulating Bcl-2 and up-regulating Bax protein expression (Haldar et al., 1994; Miyashita et al., 1994). It has been reported that BT-474 cells bear a missense mutation in the *p53* gene and show loss of heterozygosity (Bartek et al., 1990) at this locus. The effect of absence of wild-type p53 in this cell line on cell death regulation therefore needs further clarification.

In conclusion, BT-474 cells offer an interesting opportunity to investigate various aspects of growth regulation and dissemination in oestrogen-dependent breast cancer. Moreover, the capacity of this cell line to grow and metastasise in immune-deficient mice in combination with high expression of cell-surface proteins such as c-ErbB-2 and episialin make it a potentially useful *in vitro*/*in vivo* model for research in immune therapy.

**Acknowledgements**

We thank Dr JA Foekens and Professor ThJ Benraad for their help with respectively the enzyme immunoassays and ligand-binding assays. We thank Dr JHJM van Krieken and Dr B-M Ljung for their histopathological advice and NJ Kuipers-Dijkshoorn and R Keijzer for their technical assistance. This work was supported by NIH Grant No. 3 P01 CA44768-08S1 and Netherlands Cancer Foundation Grant 91-03.

**References**

ANON. (1980). EORTC Breast Cancer Cooperative Group. Revision of standards for the assessment of hormone receptors in human breast cancer: report of the Second EORTC Workshop, held on March 16–17, 1979, in The Netherlands Cancer Institute. *Eur. J. Cancer*, **16**, 1513–1515.

ALBINI A, MELCHIORI A, GAROFALO A, NOONAN DM, BASOLO F, TARA-BOLETTI G, CHADER GJ AND GAVAZZI R. (1992). Matrigel promotes retinoblastoma cell growth in vitro and in vivo. *Int. J. Cancer*, **52**, 234–240.

ARTEAGA CL, KITTEN LJ, CORONADO EB, JACOBS S, KULL Jr F, ALLRED DC AND OSBORNE CK. (1989). Blockade of the type I somatomedin receptor inhibits growth of human breast cancer cells in athymic mice. *J. Clin. Invest.*, **84**, 1418–1423.

BAO L, MATSUMURA Y, BABAN D, SUN Y AND TARIN D. (1994). Effects of inoculation site and Matrigel on growth and metastasis of human breast cancer cells. *Br. J. Cancer*, **70**, 228–232.

BARTEK J, IGGO R, GANNON J AND LANE DP. (1990). Genetic and immunochemical analysis of mutant p53 in human breast cancer cell lines. *Oncogene*, **5**, 893–899.

BISSONNETTE RP, ECHEVERRI F, MAHBOUBI A AND GREEN DR. (1992). Apoptotic cell death induced by c-myc is inhibited by bcl-2. *Nature*, **359**, 552–554.

BOISE LH, GONZÁLEZ-GARCÍA M, POSTEMA CE, DING L, LINDSTEN T, TURKA LA, MAO X, NUÑEZ G AND THOMPSON GB. (1993). *bcl-x*, a *bcl-2*-related gene that functions as a dominant regulator of apoptotic cell death. *Cell*, **74**, 597–608.

CASTLES C, FUQUA SW, KLOTZ D AND HILL S. (1993). Expression of a constitutively active estrogen receptor variant in the estrogen receptor-negative BT-20 human breast cancer cell line. *Cancer Res.*, **53**, 5934–5939.

CHUNG LW. (1991). Fibroblasts are critical determinants in prostatic cancer growth and dissemination (review). *Cancer Metastasis Rev.*, **10**, 263–274.

COLOMER R, SACEDA M, MARTIN BM, LIPPMAN ME AND LUPU R. (1992). Cross-regulation erbB-2 oncoprotein and estrogen receptor (ER) by estrogen and erbB-2 ligands (gp30/p75). *Proc. Am. Assoc. Cancer Res.*, **33**, 82.

CORNIL I, MAN S, FERNANDEZ B AND KERBEL RS. (1989). Enhanced tumorigenicity, melanogenesis, and metastases of a human malignant melanoma after subdermal implantation in nude mice. *J. Natl Cancer Inst.*, **81**, 938–944.

CORVER WE, CORNELISSE CJ AND FLEUREN GJ. (1994). Simultaneous measurement of two cellular antigens and DNA using fluorescein-isothiocyanate, R-phycoerythrin, and propidium iodide on a standard FACScan. *Cytometry*, **15**, 117–128.

DATI C, ANTONIOTTI S, TAVERNA D, PERROTEAU I AND DE BORTOLI M. (1993). Inhibition of c-erbB-2 oncogene expression by estrogens in human breast cancer cells. *Oncogene*, **5**, 1001–1006.

ELLEDGE RM, MCGUIRE WL AND OSBORNE CK. (1992). Prognostic factors in breast cancer (review). *Semin. Oncol.*, **19**, 244–253.

ELLIOT BE, TAM SP, DEXTER D AND CHEN ZQ. (1992). Capacity of adipose tissue to promote growth and metastasis of a murine mammary carcinoma: effect of estrogen and progesterone. *Int. J. Cancer*, **51**, 416–424.

EVAN GI AND LITTLEWOOD TD. (1993). The role of c-myc in cell growth (review). *Curr. Opin. Genet. Dev.*, **3**, 44–49.

FOGH J, FOGH JM AND ORFEO T. (1977). One hundred and twenty-seven cultured human tumor cell lines producing tumors in nude mice. *J. Natl Cancer Inst.*, **59**, 221–226.

FRIDMAN R, GIACCONE G, KANEMOTO T, MARTIN GR, GAZDAR AF AND MULSHINE JL. (1990). Reconstituted basement membrane (matrigel) and laminin can enhance the tumorigenicity and the drug resistance of small cell lung cancer cell lines. *Proc. Natl Acad. Sci. USA*, **87**, 6698–6702.

FRIDMAN R, KIBBEY MC, ROYCE LS, ZAIN M, SWEENEY M, JICHA DL, YANNELLI JR, MARTIN GR AND KLEINMAN HK. (1991). Enhanced tumor growth of both primary and established human and murine tumor cells in athymic mice after coinjection with Matrigel (see comments). *J. Natl Cancer Inst.*, **83**, 769–774.

FRIDMAN R, SWEENEY TM, ZAIN M, MARTIN GR AND KLEINMAN HK. (1992). Malignant transformation of NIH-3T3 cells after subcutaneous co-injection with a reconstituted basement membrane (matrigel). *Int. J. Cancer*, **51**, 740–744.

FU X, BESTERMAN JM, MONOSOV A AND HOFFMAN RM. (1991). Models of human metastatic colon cancer in nude mice orthotopically constructed by using histologically intact patient specimens. *Proc. Natl Acad. Sci. USA*, **88**, 9345–9349.

FU X, GUADAGNI F AND HOFFMAN RM. (1992). A metastatic nude-mouse model of human pancreatic cancer constructed orthotopically with histologically intact patient specimens. *Proc. Natl Acad. Sci. USA*, **89**, 5645–5649.

GILL PG, TILLEY WD, DE YOUNG NJ, LENSINK IL, DIXON PD AND HORSFALL DJ. (1991). Inhibition of T47D human breast cancer cell growth by the synthetic progestin R5020: effects of serum, estradiol, insulin, and EGF. *Breast Cancer Res. Treat.*, **20**, 53–62.

GIOVANELLA BC, STEHLIN Jr JS, WILLIAMS Jr LJ, LEE SS AND SHEPARD RC. (1978). Heterotransplantation of human cancers into nude mice: a model system for human cancer chemotherapy. *Cancer*, **42**, 2269–2281.

GRUNT T, SACEDA M, MARTIN MB AND LUPU R. (1994). The antiestrogenic effects of an erbB-2 ligand on breast cancer cell growth and on erbB-2 expression. *Proc. Am. Assoc. Cancer Res.*, **35**, 554.

HALDAR S, NEGRINI M, MONNE M, SABBIONI S AND CROCE CM. (1994). Down-regulation of *bcl-2* by *p53* in breast cancer cells. *Cancer Res.*, **54**, 2095–2097.

HANN SR AND EISENMAN RN. (1984). Proteins encoded by the human c-myc oncogene: differential expression in neoplastic cells. *Mol. Cell. Biol.*, **4**, 2486–2497.

HARWERTH IM, WELS W, SCHLEGEL J, MULLER M AND HYNES NE. (1993). Monoclonal antibodies directed to the erbB-2 receptor inhibit *in vivo* tumour cell growth. *Br. J. Cancer*, **68**, 1140–1145.

HEDLEY D, FRIEDLANDER M, TAYLOR I, RUGG C AND MUSGROVE E. (1983). Method for analysis of cellular DNA content of paraffin-embedded pathological material using flow cytometry. *J. Histochem. Cytochem.*, **31**, 1333–1335.

HORGAN K, JONES DL AND MANSEL RE. (1987). Mitogenicity of human fibroblasts in vivo for human breast cancer cells. *Br. J. Surg.*, **74**, 227–229.

HORWITZ KB. (1993). Mechanisms of hormone resistance in breast cancer. *Breast Cancer Res. Treat.*, **26**, 119–130.

HUDZIAK RM, LEWIS GD, SHALABY MR, EESSALU TE, AGGARWAL BB, ULLRICH A AND SHEPARD HM. (1988). Amplified expression of the HER2/ERBB2 oncogene induces resistance to tumor necrosis factor alpha in NIH 3T3 cells. *Proc. Natl Acad. Sci. USA*, **85**, 5102–5106.

HYNES N. (1993). Amplification and overexpression of the erbB-2 gene in human tumors: its involvement in tumor development, significance as a prognostic factor, and potential as a target for cancer therapy. *Semin. Cancer Biol.*, **4**, 19–26.

IGNAR-TROWBRIDGE DM, NELSON KG, BIDWELL MC, CURTIS SW, WASHBURN TF, MCLACHLAN JA AND KORACH KS. (1992). Coupling of dual signaling pathways: epidermal growth factor action involves the estrogen receptor. *Proc. Natl Acad. Sci. USA*, **89**, 4658–4662.

KAMESAKI S, KAMESAKI H, JORGENSEN TJ, TANIZAWA A, POMMIER Y AND COSSMAN J. (1993). bcl-2 protein inhibits etoposide-induced apoptosis through its effects on events subsequent to topoisomerase II-induced DNA strand breaks and their repair. *Cancer Res.*, **53**, 4251–4256.

KOGA M, MUSGROVE E AND SUTHERLAND R. (1990). Differential effects of phorbol ester on epidermal growth factor receptors in estrogen receptor-positive and -negative breast cancer cell lines. *Cancer Res.*, **50**, 4849–4855.

KYPRIANOU N, ENGLISH HF, DAVIDSON NE AND ISAACS JT. (1991). Programmed cell death during regression of the MCF-7 human breast cancer following estrogen ablation. *Cancer Res.*, **51**, 162–166.

LASFARGUES EY, COUTINHO WG AND REDFIELD ES. (1978). Isolation of two human tumor epithelial cell lines from solid breast carcinomas. *J. Natl Cancer Inst.*, **61**, 967–978.

LEUNG CK AND SHIU RP. (1981). Required presence of both estrogen and pituitary factors for the growth of human breast cancer cells in athymic nude mice. *Cancer Res.*, **41**, 546–551.

LICHTENSTEIN A, BERENSON J, GERA JF, WALDBURGER K, MARTINEZ-MAZA O AND BEREK JS. (1993). Resistance of human ovarian cancer cells to tumor necrosis factor and lymphokine-activated killer cells: correlation with expression of HER2/neu oncogenes. *Cancer Res.*, **50**, 7364–7370.

LONG B, MCKIBBEN BM, LYNCH M AND VAN DEN BERG HW. (1992). Changes in epidermal growth factor receptor expression and response to ligand associated with acquired tamoxifen resistance or oestrogen independence in the ZR-75-1 human breast cancer cell line. *Br. J. Cancer*, **65**, 865–869.

LOWE SW, SCHMITT EM, SMITH SW, OSBORNE BA AND JACKS T. (1993). p53 is required for radiation-induced apoptosis in mouse thymocytes (see comments). *Nature*, **362**, 847–849.

LUPU R AND LIPPMAN M. (1993). The role of erbB2 signal transduction pathways in human breast cancer. *Breast Cancer Res. Treat.*m **27**, 83–93.

MEHTA RR, GRAVES JM, HART GD, SHILKAITIS A AND DAS GUPTA TK. (1993). Growth and metastasis of human breast carcinomas with Matrigel in athymic mice. *Breast Cancer Res. Treat.*, **25**, 65–71.

MILLER FR AND MCINERNEY D. (1988). Epithelial component of host–tumor interactions in the orthotopic site preference of a mouse mammary tumor. *Cancer Res.*, **48**, 3698–3701.

MILLER FR, MEDINA D AND HEPPNER GH. (1981). Preferential growth of mammary tumors in intact mammary fatpads. *Cancer Res.*, **41**, 3863–3867.

MIYASHITA T, KRAJEWSKI S, KRAJEWSKA M, WANG HG, LIN HK, LIEBERMANN DA, HOFFMAN B AND REED JC. (1994). Tumor suppressor p53 is a regulator of *bcl-2* and *bax* gene expression in vitro and in vivo. *Oncogene*, **9**, 1799–1805.

MURPHY LC AND DOTZLAW H. (1989). Endogenous growth factor expression in T-47D, human breast cancer cells, associated with reduced sensitivity to antiproliferative effects of progestins and antiestrogens. *Cancer Res.*, **49**, 599–604.

NIEHANS GA, SINGLETON TP, DYKOSKI D AND KIANG DT. (1993). Stability of HER-2/neu expression over time and at multiple metastatic sites. *J. Natl Cancer Inst.*, **85**, 1230–1235.

NOEL A, SIMON N, RAUS J AND FOIDART JM. (1992). Basement membrane components (matrigel) promote the tumorigenicity of human breast adenocarcinoma MCF7 cells and provide an in vivo model to assess the responsiveness of cells to estrogen. *Biochem. Pharmacol.*, **43**, 1263–1267.

OLTVAI ZN, MILLIMAN L AND KORSMEYER SJ. (1993). Bcl-2 heterodimerizes in vivo with a conserved homolog, Bax, that accelerates programmed cell death. *Cell*, **74**, 609–619.

PASSANITI A, ISAACS JT, HANEY JA, ADLER SW, CUJDIK TJ, LONG PV AND KLEINMAN HK. (1992). Stimulation of human prostatic carcinoma tumor growth in athymic mice and control of migration in culture by extracellular matrix. *Int. J. Cancer*, **51**, 318–324.

PAVELIC ZP, PAVELIC L, LOWER EE, GAPANY M, GAPANY S, BARKER FA AND PREISLER HD. (1992). c-myc, c-erbB-2, and Ki-67 expression in normal breast tissue and in invasive and noninvasive breast carcinoma. *Cancer Res.*, **52**, 2597-2602.

PRETLOW TG, DELMORO CM, DILLEY GG, SPADAFORA CG AND PRETLOW TP. (1991). Transplantation of human prostatic carcinoma into nude mice in Matrigel. *Cancer Res.*, **51**, 3814-3817.

PRICE J, POLYZOS A, RUO DAN ZHANG AND DANIELS L. (1990). Tumorgenicity and metastasis of human breast carcinoma cell lines in nude mice. *Cancer Res.*, **50**, 717-721.

READ LD, KEITH Jr D, SLAMON DJ AND KATZENELLENBOGEN BS. (1993). Hormonal modulation of HER-2/neu protooncogene messenger ribonucleic acid and p185 protein expression in human breast cancer cell lines. *Cancer Res.*, **50**, 3947-3957.

REED J. (1994). Mini-review: cellular mechanisms of disease series. Bcl-2 and the regulation of the programmed cell death. *J. Cell Biol.*, **124**, 1-6.

SEBESTENY A, TAYLOR-PAPADIMITRIOU J, CERIANI R, MILLIS R, SCHMITT C AND TREVAN D. (1979). Primary human breast carcinomas transplantable in the nude mouse. *J. Natl Cancer Inst.*, **63**, 1331-1337.

SHAFIE SM AND LIOTTA LA. (1980). Formation of metastasis by human breast carcinoma cells (MCF-7) in nude mice. *Cancer Lett.*, **11**, 81-87.

STERLING-LEVIS K, WHITE L, TRICKETT AE, GRAMACHO C, PITTMAN SM AND TOBIAS V. (1993). Heterotransplantation of early B-lineage acute lymphoblastic leukemia using a solubilized attachment matrix (Matrigel). *Cancer Res.*, **53**, 1222-1225.

TOPLEY P, JENKINS DC, JESSUP EA AND STABLES JN. (1993). Effect of reconstituted basement membrane components on the growth of a panel of human tumour cell lines in nude mice. *Br. J. Cancer*, **67**, 953-958.

TSUNODA R, NAKAYAMA M, HEINEN E, MIYAKE K, SUZUKI K, SUGAI N AND KOJIMA M. (1992). Emperipolesis of lymphoid cells by human follicular dendritic cells in vitro. *Virchows Arch. B Cell Pathol.*, **62**, 69-78.

VINDELOV L, CHRISTENSEN I AND NISSEN N. (1983). A detergent-trypsin method for the preparation of nuclei for flow cytometric DNA analysis. *Cytometry*, **3**, 323-327.

WARRI AM, LAINE AM, MAJASUO KE, ALITALO KK AND HARKONEN PL. (1991). Estrogen suppression of erbB2 expression is associated with increased growth rate of ZR-75-1 human breast cancer cells in vitro and in nude mice. *Int. J. Cancer*, **49**, 616-623.

WECKBECKER G, LIU R, TOLCSVAI L AND BRUNS C. (1992). Antiproliferative effects of somatostatin analogue octreotide (SMS 201-995) on ZR-75-1 human breat cancer cells in vivo and in vitro. *Cancer Res.*, **52**, 4973-4978.

WHITE AC, LEVY JA AND MCGRATH CM. (1982). Site-selective growth of a hormone-responsive human breast carcinoma in athymic mice. *Cancer Res.*, **42**, 906-912.

WIJSMAN J, JONKER R, KEIJZER R, VAN DE VELDE CH, CORNELISSE C AND VAN DIERENDONCK J. (1993). A new method to detect apoptosis in paraffin sections: in situ end-labelling of fragmented DNA. *J. Histochem. Cytochem.*, **41**, 7-12.

WILTSCHKE C, TYL E, SPEISER P, STEININGER A, ZEILLINGER R, KURY F, CZERWENKA K, KUBISTA E, PREIS P, KRAINER M AND ZIELINSKI C. (1994). Increased natural killer cell activity correlates with low or negative expression of the HER-2/neu oncogene in patients with breast cancer. *Cancer*, **73**, 135-139.

YUE W AND BRODIE A. (1993). MCF-7 human breast carcinomas in nude mice as a model for evaluation aromatase inhibitors. *J. Steroid Biochem. Mol. Biol.*, **44**, 671-673.

ZHU Y-M, BRADBURY DA AND RUSSELL NH. (1994). Wild-type p53 is required for apoptosis induced by growth factor deprivation in factor-dependent leukaemic cells. *Br. J. Cancer*, **69**, 468-472.